# Nanoporous Carbon Materials Derived from Washnut Seed with Enhanced Supercapacitance

**DOI:** 10.3390/ma13102371

**Published:** 2020-05-21

**Authors:** Ram Lal Shrestha, Timila Shrestha, Birendra Man Tamrakar, Rekha Goswami Shrestha, Subrata Maji, Katsuhiko Ariga, Lok Kumar Shrestha

**Affiliations:** 1 Department of Chemistry, Amrit Campus, Tribhuvan University, Kathmandu 44613, Nepal; swagatstha@gmail.com (R.L.S.); timilastha@gmail.com (T.S.); 2 Department of Chemistry, Tri-Chandra Multiple Campus, Tribhuvan University, Kathmandu 44600, Nepal; tamrakar_birendra@hotmail.com; 3 International Center for Materials Nanoarchitectonics (WPI-MANA), National Institute for Materials Science (NIMS), 1-1 Namiki, Ibaraki 305-0044, Japan; MAJI.Subrata@nims.go.jp (S.M.); ARIGA.Katsuhiko@nims.go.jp (K.A.); 4 Graduate School of Frontier Sciences, The University of Tokyo, 5-1-5 Kashiwanoha, Kashiwa, Chiba 277-8561, Japan

**Keywords:** Washnut seed, chemical activation, micro/mesoporous carbon, supercapacitor

## Abstract

Nanoporous activated carbons-derived from agro-waste have been useful as suitable and scalable low-cost electrode materials in supercapacitors applications because of their better surface area and porosity compared to the commercial activated carbons. In this paper, the production of nanoporous carbons by zinc chloride activation of Washnut seed at different temperatures (400–1000 °C) and their electrochemical supercapacitance performances in aqueous electrolyte (1 M H_2_SO_4_) are reported. The prepared nanoporous carbon materials exhibit hierarchical micro- and meso-pore architectures. The surface area and porosity increase with the carbonization temperature and achieved the highest values at 800 °C. The surface area was found in the range of 922–1309 m^2^ g^−1^. Similarly, pore volume was found in the range of 0.577–0.789 cm^3^ g^−1^. The optimal sample obtained at 800 °C showed excellent electrochemical energy storage supercapacitance performance. Specific capacitance of the electrode was calculated 225.1 F g^−1^ at a low current density of 1 A g^−1^. An observed 69.6% capacitance retention at 20 A g^−1^ indicates a high-rate capability of the electrode materials. The cycling stability test up to 10,000 cycles revealed the outstanding stability of 98%. The fascinating surface textural properties with outstanding electrochemical performance reveal that Washnut seed would be a feasible agro-waste precursor to prepare nanoporous carbon materials as a low-cost and scalable supercapacitor electrode.

## 1. Introduction

Supercapacitors or electrical double-layer capacitors (EDLC), the most convenient and state-of-the-art electrochemical energy storage systems, with outstanding power density (>400 kW kg^−1^), unusually long cycle stability (>10,000), rapid charging-discharging with enhanced rate capability and poor internal resistance, and environmentally friendly and low-cost, have been extensively used for high power electronic devices [1,2,3,4,5,6,7,8,9,10]. In EDLCs, electrolyte ions are adsorbed at the electrode surface by fast dynamic propagation and form double layers of electrical charges [11]. Supercapacitors offer greater power densities compared to the traditional batteries and fuel cells; therefore, they have been implemented in industrial power and energy management systems [12,13]. However, compared to batteries, supercapacitors exhibit low energy density, and hence, they have explored less in the potential technological applications [14,15]. The energy density of supercapacitors is based on the specific capacitance (*C_s_*) and the real operating potential window (*V*). The specific capacitance directly depends on the properties of the materials used for the electrode and potential window depends on the electrolyte used for the construction of the supercapacitor cells [16,17]. Since the operating potential window is fixed in aqueous electrolyte (~1.2 V), it is important to improve the structure and properties of the materials to be used as supercapacitor electrodes so that introducing the novel and smart electrode materials could exhibit high specific capacitances and also induces good combination with electrolyte [18,19]. It is important to have a good match between electrolyte ions and pore size of the electrode material, and wettability of electrode and electrolyte conductivity [20].

Recently, nanoporous activated carbons have received considerable interest as the leading supercapacitor electrode materials because of the low production cost, outstanding cycle stability, and excellent surface specific surface area and porosity [21,22,23,24,25]. Different porous carbon materials have been produced either from synthetic carbon sources or natural biomasses by direct carbonization or chemical activation methods as well as template method and explored as supercapacitor electrode materials [26,27,28,29,30]. Of several methods, natural biomass or agricultural wastes-derived nanoporous carbons by physical and chemical activation methods represent the feasible, scalable, and low-cost method. Biomass-derived nanoporous activated carbons exhibit very high surface area and offer large porosity because of their unique hierarchical micro- and meso-porous architectures, and also have good electrical conductivity and excellent electrochemical stability, which are highly desired in the emerging electrochemical energy storage supercapacitors applications [31,32,33].

All the natural biomasses or agricultural wastes are lignocellulose materials containing cellulose, hemicellulose and lignin, which upon pyrolysis at lower temperatures (200–300 °C) under the air or nitrogen gas atmosphere produces biochar with a low specific surface area and low porosity. Therefore, the biochar is not useful in supercapacitors. Nevertheless, the biochar can be activated and transformed into high surface area hierarchical nanoporous carbon materials with well-developed porosity desired in supercapacitors by the direct carbonization and physical or chemical activation methods [34,35]. The industrial-scale manufacture of nanoporous activated carbons is based on the physical activation of the biochar at higher temperatures (800–1100 °C) under the flow of steam/or carbon dioxide. This is a simple and cost-effective fabrication process. Physically activated carbons exhibit specific surface area in the range of 500–1000 m^2^ g^−1^. The surface area of the biomass-derived physically activated carbons can further be enhanced, which can be enabled by the chemical activation method and the resulting carbon materials achieve a surface area far more than 1000 m^2^ g^−1^ [36]. The chemical activation method includes the impregnation of biomass or biochar with an activating agent and then the mixture is carbonized in the temperature ranges of 400–1000 °C in an inert atmosphere of nitrogen or argon gas [37]. Activating agents generally include dehydrating salts, such as zinc chloride (ZnCl_2_), sodium carbonate (Na_2_CO_3_), and also acid and alkali. Lignocellulose undergoes pyrolytic decomposition upon mixing with these activating agents and porosity enhancement can be achieved as a result of the depolymerization and dehydration of the biochar. Of several activating reagents, ZnCl_2_ is a widely used chemical activating agent, which dehydrates and accelerates the decomposition of carbonaceous materials during the carbonization process and also restricts the formation of tar giving a high yield carbon. ZnCl_2_ contributes to creating a porous structure acting as a template; intercalated ZnCl_2_ upon washing creates the void space in the carbon matrix. Recently, using various agricultural wastes or biomass such as rice husks [38], corncob [39,40], pistachio shell [41], pitch [42], bamboo [43], Batata leaves and stalks [44], Peanut dregs [45], Lapsi seed (*Choerospondias axillaris*) [46], etc. high surface area nanoporous carbons with large porosity, interconnected mesopores and uniform pore size distribution essentially required in supercapacitor applications have been produced.

In this paper, the synthesis of nanoporous activated carbons from Washnut seed agro-waste by the ZnCl_2_ activation method and their electrochemical supercapacitance performance are reported. Electrochemical measurements were carried out on three-electrode cells in 1 M H_2_SO_4_. Pre-carbonized Washnut seed powder (char) was impregnated with the activating agent, ZnCl_2_ at 1:1 weight ratio, and carbonizations were carried out at different temperatures from 400 to 1000 °C. ZnCl_2_-activated Washnut seed-derived nanoporous carbons display a hierarchical porous architecture containing both the micro- and meso-porous and offer a high specific surface area (1309 m^2^ g^−1^) and pore volume (0.789 cm^3^ g^−1^). An increase in carbonization temperature increases the surface area giving the best surface textural properties at 800 °C. The working electrode prepared with the optimal carbon sample showed excellent supercapacitance performance with the high specific capacitance calculated 225.1 F g^−1^ at 1 A g^−1^. The electrode sustained 69.6% capacitance retention at a high current density of 20 A g^−1^ showing the high-rate capability of the electrode. Furthermore, an outstanding cycling stability of 98% was recorded after 10,000 charging–discharging cycles demonstrating that Washnut seed could be an appropriate alternative low-cost biomass for the production scalable carbon electrodes for high-performance supercapacitors.

## 2. Materials and Methods

### 2.1. Preparation of Nanoporous Activated Carbons

Agro-waste Washnut seed was washed with distilled water, dried at 80 °C for 6 h and ground into the powder form in a mechanical crusher. The Washnut seed powder was pre-carbonized at 300 °C in air for 3 h. Pre-carbonized Washnut seed powder was mixed with ZnCl_2_, a chemical activating agent at 1:1 weight ratio and carbonizations were carried out at different temperatures (400–1000 °C) under the nitrogen flux (120 cc min^−1^) in a tube furnace (KOYO, Tokyo, Japan). Temperature ramp and hold time was set to 5 °C min^−1^ and 3 h, respectively. The obtained nanoporous carbon samples were treated with a dilute hydrochloric acid solution (0.5 M HCl) for removing the unreacted zinc chloride followed by distilled water washing (several times). The product was dried in vacuum at 80 °C for 6 h and further grounded into fine powders, and sieved through 250 μm mesh size. The obtained samples were referred to as WNC_400, WNC_600, WNC_800, and WNC_1000 depending on the carbonization temperature. For comparison, Washnut seed powder was also directly carbonized at 800 °C without an activating agent and the product is referred to as WNP_800.

### 2.2. Characterizations

Washnut seed-derived nanoporous carbon materials were subjected to advanced characterizations. Surface morphology and the pore structure were studied by scanning electron microscopy (SEM: S-4800, Hitachi Co., Ltd. Tokyo, Japan). The S-4800 was operated at an operating voltage of 10 kV and a field emission current of 10 μA. SEM samples were platinum-coated (~2 nm) on a Hitachi S-2030 ion-coater, to avoid sample charging effects. For the structural determination, powder X-ray diffraction (XRD) patterns were recorded on a Rigaku X-ray diffractometer, RINT, Tokyo, Japan, operating the X-ray diffractometer at 25 °C in the range 10 to 50° at 40 kV and 40 mA with Cu-K_α_ radiation. Graphitization and defects of the carbon samples were studied by Raman scattering (Jobin-Yvon T64000 Raman spectrometer, Edison, NJ, USA). Sample on glass substrate were excited with a green laser (514.5 nm) at 0.01 mW power and exposed for 30 s. Fourier-transformed infrared (FTIR) spectra were recorded by KBr pellet method on a Nicolet 4700 (Thermo Electron Corporation, Walthan, MA, USA) at 25 °C. For the determination of surface textural properties (surface areas, pore volumes, average pore sizes, and pore size distributions, nitrogen adsorption/desorption isotherms were measured using on Quantachrome Autosorb-iQ2, Boynton Beach, FL, USA: an automatic adsorption instrument. Carbon sample (~20 mg) was degassed at 120 °C for 24 h before measurements and isotherms were recorded at liquid nitrogen temperature 77.35 K. From the sorption isotherms, Brunauer–Emmett–Teller (BET) surface area, and pore size distributions were determined by Barrett–Joyner–Halenda (BJH: for mesopore) method and density functional theory (DFT: for micropore).

### 2.3. Electrochemical Measurements

Cyclic voltammetry (CV) and galvanostatic charge-discharge (CD) measurements were carried out in a three-electrode system in an aqueous (1 M H_2_SO_4_) solution at 25 °C to study the electrochemical supercapacitance performances of the Washnut-derived nanoporous activated carbons. Modified glassy carbon electrode (GCE, ALS Co., Ltd, Tokyo, Japan) was used as the working electrode. For the working electrode preparation, the GCE was mirror polished with alumina (Al_2_O_3_) slurry, sonicated in acetone for 10 min and cleaned with distilled water. The prepared carbon material was dispersed in water-ethanol (4:1) mixture (1 mg mL^−1^) by sonicating the mixture for 45 min in a bath sonicator (BRANSON 3510, Hampton, NH, USA). The obtained suspension (3 µL) was dropcast onto the GCE and dried at 60 °C for 6 h to evaporate the solvents. Nafion solution (5 µL: 5% in ethanol) was then added on top of the carbon materials on the GCE surface as a binder and dried at 80 °C in vacuum for 12 h before the electrochemical measurements. The CV and CD measurements were carried out on a CHI 850D work station (CH Instruments, Inc. Austin, TX, USA) using a platinum wire was used as a counter and Ag/AgCl as the reference electrode.

From CD profiles, specific capacitance was calculated as:

(1) $$C_{s}=\frac{I\cdot t}{m\cdot ∆V}$$

where *C*_s_, *I*, *t*, *m*, and ∆*V*, respectively, represent the specific capacitance (F g^−1^), discharge current (A), discharge time (s), mass of active electrode materials (g), and the operating potential window (*V*_final_–*V*_initial_).

## 3. Results and Discussion

**Figure 1:**
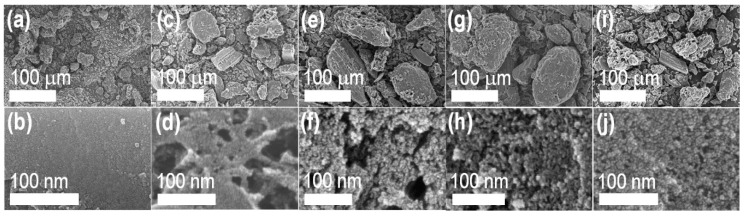
SEM images of Washnut seed-derived nanoporous activated carbons: (**a**,**b**) WNP_800; (**c**,**d**) WNC_400; (**e**,**f**) WNC_600; (**g**,**h**) WNC_800; (**i**,**j**) WNC_1000.

Surface morphology and pore architecture of the ZnCl_2_ activated Washnut seed-derived nanoporous carbon materials were studied by scanning electron microscopy (SEM) imaging. Figure 1 depicts typical SEM images of the activated samples WNC_400, WNC_600, WNC_800, and WNC_1000 both at low- and high-magnification modes. For comparison, SEM images of a directly carbonized sample (WNP_800) without ZnCl_2_ is also included. The SEM images at low magnification (Figure 1a,c,e,g,i) reveal the Washnut seed-derived carbons have irregular morphology with micron-size particles containing visible microporous surface structure. In the high-magnification SEM images (Figure 1b,d,f,h,j), the chemically activated carbon surface display hierarchical micro- and meso-pore architectures. While in the directly carbonized WNP_800 sample, a significantly smaller number of visible micro/mesopores is observed. A marco-sized interconnected channel like surface morphology can be visible in the high-magnification images of WNC_400 (Figure 1d), and WNC_600 (Figure 1f). On the other hand, a rather uniform mesoporous surface with interconnected pore structure can be seen in the high-magnification SEM images of WNC_800 (Figure 1h), and WNC_1000 (Figure 1j).

**Figure 2:**
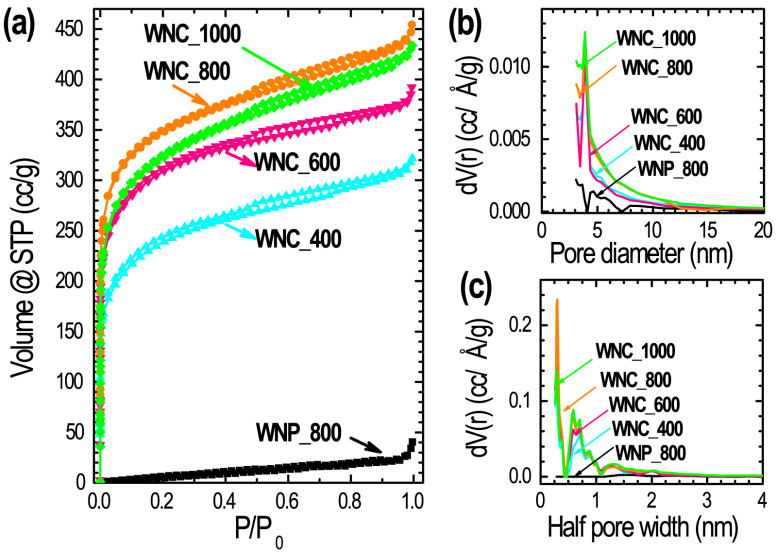
(**a**) Nitrogen sorption (adsorption/desorption) isotherms of WNP_800, WNC_400, WNC_600, WNC_800, and WNC_1000 measured at 77 K, and the pore size distributions profiles obtained by (**b**) Barrett–Joyner–Halenda (BJH) method and (**c**) density functional theory (DFT) method.

Specific surface area, pore volumes, pore size distribution and average pore size were detected using nitrogen adsorption/desorption analysis. Figure 2a shows the nitrogen sorption isotherms of WNP_800, WNC_400, WNC_600, WNC_800, and WNC_1000 measured at 77 K and Figure 2b,c show the pore size distributions estimated from the BJH and DFT method, respectively. The sorption isotherms reveal that all the chemically activated samples exhibit mixed Type-I/Type-IV isotherms, indicating the hierarchical micro- and meso-porous characteristics of the samples [47]. The strong nitrogen adsorption of chemically activated samples at lower relative pressure (P/P_0_ < 0.03) is the indication of plenty of micropores. While the hysteresis loops at higher relative pressures suggests that the samples also contain mesopore structure. Unlike the chemically activated samples, the sorption isotherm of the directly carbonized sample, WNP_800, exhibits Type-III isotherm suggesting nonporous or microporous characteristics of the sample. The pore size distributions profiles (Figure 2b: obtained by BJH analysis; Figure 2c: obtained by DFT analysis) further confirm the presence of hierarchical micro- and meso-porous structures in the activated samples, WNC_400, WNC_600, WNC_800, and WNC_1000. The average micro- and meso-pore diameters are calculated 0.57 and 3.88 nm, respectively. These micro- and meso-pore distributions over the prepared carbon materials provide a sufficiently large surface area, where electrolyte ions can be adsorbed and also promotes the fast and easy diffusion in the electrode surface required in high energy storage systems [48].

Due to the lack of well-developed porosity, specific surface area of the directly carbonized sample WNP_800 is low (39.2 m^2^ g^−1^). As a result, low energy storage performance is expected compared to the ZnCl_2_ activated samples. The surface textural properties of the prepared samples obtained from the nitrogen sorption analysis are presented in Table 1.

**Figure 3:**
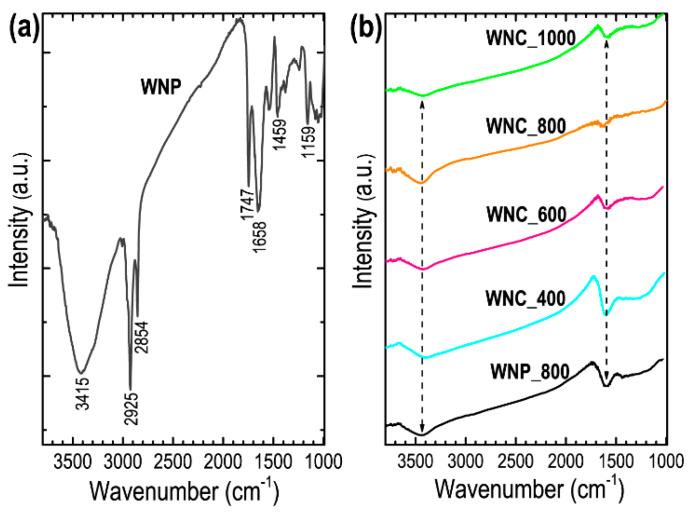
(**a**) FTIR spectrum of Washnut seed powder (WNP) before activation, and (**b**) FTIR spectra of carbonized/activated samples WNP_800, WNC_400, WNC_600, WNC_800, and WNC_1000 measured at 25 °C.

Figure 3a shows the FTIR spectrum of Washnut seed powder precursor materials before pre-carbonization or ZnCl_2_ activation. As expected, the precursor contains oxygen-containing functional groups (–OH, C=O, COOH, ether, phenol and lactones) [33,38,49]. Some of these functional groups sustain even after carbonization at high temperature. FTIR spectra of the carbon samples display a broad peak centered nearly at 3435 cm^−1^ (Figure 3b), which can be attributed to the O−H functional groups and a weak FTIR peak approximately at 1634 cm^-1^ come from the aromatic C=C stretching vibration commonly observed in the activated carbons [38,49].

**Figure 4:**
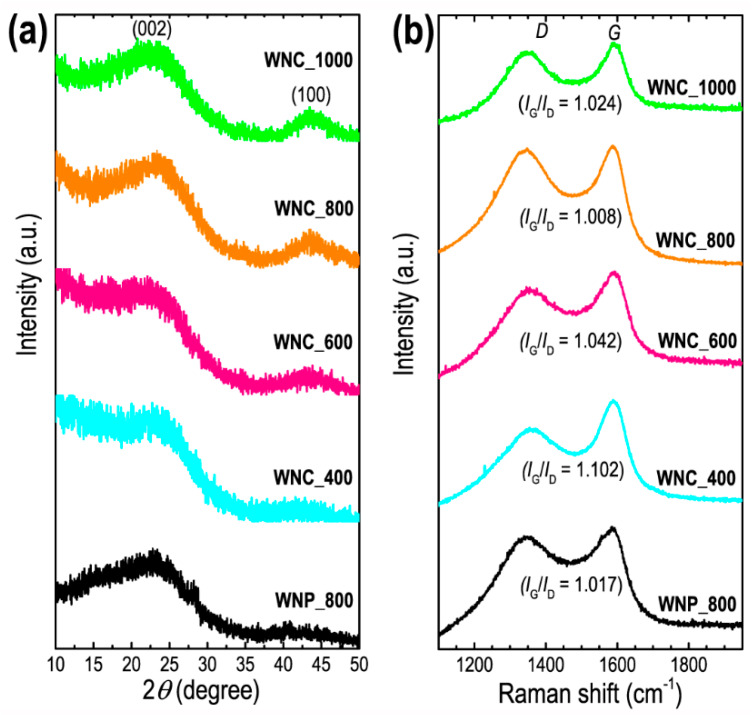
(**a**) Powder X-ray diffraction (pXRD) patterns of WNP_800, WNC_400, WNC_600, WNC_800, and WNC_1000 recorded at 25 °C, and (**b**) Raman scattering spectra of WNP_800, WNC_400, WNC_600, WNC_800, and WNC_1000 recorded at 25 °C.

Powder X-ray diffraction (pXRD) and Raman scattering and analysis were also carried out to further evaluate the structural features of the Washnut seed-derived nanoporous carbon materials. Figure 4 shows the pXRD patterns and Raman scattering spectra of the prepared carbon samples.

The pXRD patterns (Figure 4a) display characteristics of amorphous carbon with two broad peaks approximately at ~23 and 43° equivalent to the (002) and (100) planes observed in graphitic carbon particles. Broad (002) diffraction profile at a diffraction angle of ~23° indicates that the Washnut seed-derived nanoporous carbon materials have poorly ordered amorphous structure and also smaller graphitic clusters of the samples [50]. Raman spectra (Figure 4b) of all the samples exhibit *D* and *G* bands approximately at ~1350 and ~1588 cm^−1^, respectively [50]. The *D* band corresponds to the disordered structure of carbon induced by defects and impurities, while *G* band corresponds to the ordered graphitic layer structure. The presence of obvious *D* and *G* bands confirms the formation of activated carbons. The intensity ratio of *G* and *D* Raman bands (*I*_G_/*I*_D_) measures the graphitization degree of the carbons. The *I*_G_/*I*_D_ ratio is found in the range of 1.008 to 1.1042 characteristics of graphitic carbons commonly observed in activated carbon materials with a low degree of defects [50,51].

**Figure 5:**
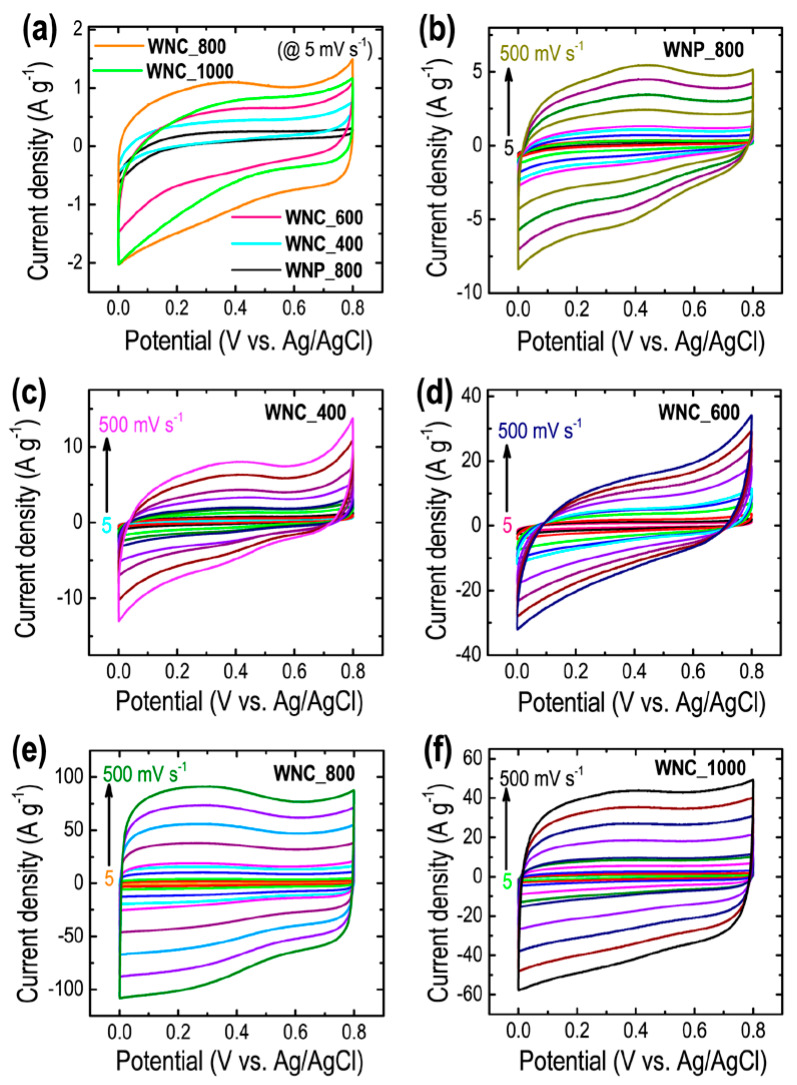
(**a**) Comparison of cyclic voltammetry (CV) profiles of all the prepared carbons at 5 mV s^−1^, and the CV profile vs. scan rates. (**b**) WNC_800, (**c**) WNC_400, (**d**) WNC_600, (**e**) WNC_800, and (**f**) WNC_1000.

Electrochemical supercapacitance performance of the prepared activated nanoporous carbon materials was performed in a three-electrode system in aqueous electrolyte (1 M H_2_SO_4_). Cyclic voltammetry (CV) profiles were measured in a wide scan rates of 5 to 500 mV s^−1^ and galvanostatic charge-discharge (CD) curves were recorded at from 1 to 20 A g^−1^ at 25 °C. Figure 5a compares the CV profiles of WNP_800, WNC_400, WNC_600, WNC_800, and WNC_1000 at 5 mV s^−1^ as typical example.

As can be seen in Figure 5a, all the CV profiles exhibit quasi-rectangular shape characteristics of the EDLC behavior [50,52]. Depending on the carbon sample, the current differs. The lowest current of the directly carbonized sample, WNP_800, shows the poor supercapacitance performance of the materials, which can be attributed to the poor surface textural properties (surface area and porosity). The current collection under the CV profile increases with temperature up to 800 °C, i.e., from WNC_400 to WNC_800 sample and declines slightly in the WNC_1000, which is directly correlated to the surface area and porosity of the carbon samples (see Table 1). Due to the highest surface area (plenty of micropores), the WNC_800 electrode offers a large surface area for the adsorption of electrolyte ions. Furthermore, due to large porosity, and interconnected hierarchical porous structure, the electrode also offers an easy path and fast diffusion of the electrolyte ion to the electrode surface and thus showed excellent energy storage capacity. Figure 5b–f shows the CV profiles of directly carbonized sample WNP_800, and ZnCl_2_ activated samples WNC_400, WNC_600, WNC_800, and WNC_1000 at different scan rates from 5 to 500 mV s^−1^. The area under the CV profiles increases with scan rate sustaining the semi-rectangular shape of the CV curve even at the high scan rate of 500 mV s^−1^ indicating the fast electrolyte ions diffusion [46,47,48,50].

**Figure 6:**
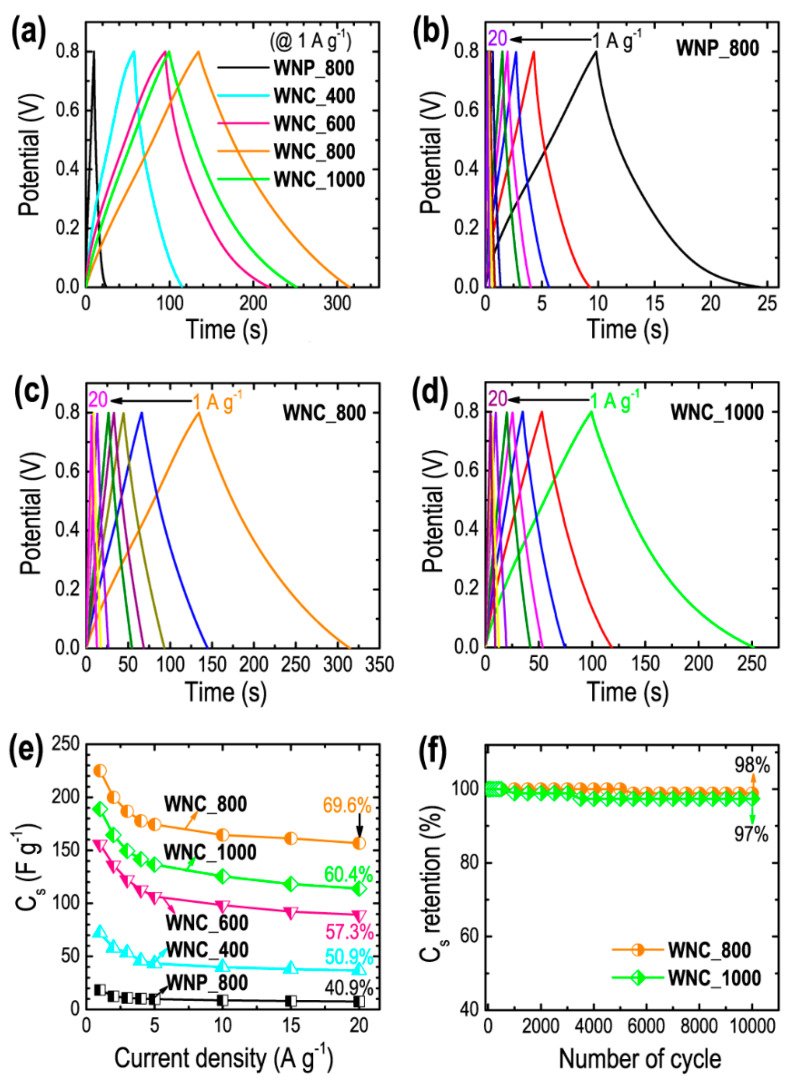
(**a**) Charge-discharge (CD) curves of WNP_800, WNC_400, WNC_600, WNC_800, and WNC_1000 at a constant current density of 1 A g^−1^, CD profiles vs. current density for (**b**) WNP_800, (**c**) WNC_800, and (**d**) WNC_100 as typical examples, (**e**) specific capacitance (*C*_s_) vs. current density, and (**g**) cycling stability performances of the WNC_800 and WNC_1000 electrodes for 10,000 cycles as typical example.

The electrochemical supercapacitance performances of the WNP_800, WNC_400, WNC_600, WNC_800, and WNC_1000 was also studied performing galvanostatic charge-discharge (CD) measurements over a wide range of current densities (1 to 20 A g^−1^). As seen in Figure 6a, triangular shape CD curves, which indicate the EDLC behavior of the electrode material are commonly observed in the CD profiles of WNP_800, WNC_400, WNC_600, WNC_800, and WNC_1000 electrodes at 1 A g^−1^ [50,53]. Here again, the CD profile of the optimal sample, WNC_800, has the longest discharging time suggesting the maximum energy storage capacity compared to the electrodes. Using Equation (1), the C_S_ measurements were calculated 18.3 F g^−1^ (WNP_800), 71.9 F g^−1^ (WNC_400), 155.8 F g^−1^ (WNC_600), 225.1 F g^−1^ (WNC_800), and 188.7 F g^−1^ (WNC_1000), which is highly interrelated with the porosity of the materials; the higher the microporosity the better the energy storage capacity (see Table 1). In Figure 6b–d, the CD profiles vs. current densities of selected samples are shown as typical examples. All the CD profiles represent characteristics of the EDLC and sustain high capacity even at 20 A g^−1^, a high current density. All the chemically activated nanoporous carbon materials display more than 50% capacitance retention achieving outstanding 69.6% for the optimal sample (Figure 6e), which demonstrates the high rate capability of the electrode material required in supercapacitor devices. Furthermore, cycling stability tested for the 10,000 charging-discharging cycles revealed outstanding stability with a very low capacitance loss of about 2%–3% (Figure 6f) indicating that the Washnut derived-nanoporous carbons have a huge potential and can be explored as supercapacitor electrodes [54,55].

The specific capacitance of the Washnut-derived carbon electrode is comparable with the specific capacitances of other nanoporous carbon materials prepared from different biomass (Table 2).

The obtained specific capacitance of 225.1 F g^−1^ is not sufficient for the design of high energy density advanced supercapacitors [32,33,48,55]. Several previous examples have demonstrated that the electrochemical supercapacitive performance of the nanoporous carbon materials depends on the various important parameters such as surface area, porosity, pore size distribution, the hierarchy on the pore (micro/mesopore) architecture, and interconnectivity of the mesopores for easy electrolyte ion diffusion, conductivity, and wettability of the electrode surface [48]. Tailoring micro-porosity, nitrogen-doping, and composite preparation with pseudocapacitive metal oxide nanoparticles would offer effective strategies to enhance the supercapacitance of biomass-derived nanoporous carbons to address the social demands.

## 4. Conclusions

In conclusion, nanoporous activated carbon materials have been prepared by ZnCl_2_ activation of Washnut seed powder and their electrochemical supercapacitance performances have been investigated in an aqueous electrolyte (1 M H_2_SO_4_) on a three-electrode cell. Specific surface area and pore volume were found in the ranges 922–1309 m^2^ g^−1^ and 0.577–0.789 cm^3^ g^−1^, respectively, depending on carbonization temperatures. Because of the excellent textural properties including high surface areas, well-developed porosity, and bimodal micro- and meso-pore architecture with graphitic pore walls, the Washnut seed-derived nanoporous activated carbons display outstanding supercapacitance such that the electrode achieved a high specific capacitance of 225.1 F g^−1^ at a current density of 1 A g^−1^ and retained a high rate capability of 69.6% at 20 A g^−1^. Furthermore, the electrode showed excellent cycling stability sustaining 98% capacity retention even after 10,000 charge-discharge cycles. Therefore, it can be concluded that as an agricultural waste, Washnut seed denotes a suitable biomass for the scalable production of high surface area and large porosity carbons essentially desired as the electrode materials for high-performance supercapacitors.

## Figures and Tables

**Table 1:** Surface textural properties of the Washnut seed-derived nanoporous activated carbons.

Carbon Sample	*SSA* (m^2^ g^−1^)	*S*_micro_ (m^2^ g^−1^)	*S*_meso_ (m^2^ g^−1^)	*V*_p_ (cm^3^ g^−1^)	*V*_micro_ (cm^3^ g^−1^)	*D*_meso_ (nm)	*D*_micro_ (nm)
WNP_800	39.2	15.3	23.9	0.099	0.037	3.09	−
WNC_400	922.4	836.5	85.9	0.577	0.444	3.88	0.573
WNC_600	1157.6	1080.5	77.1	0.662	0.535	3.88	0.548
WNC_800	1309.8	1196.1	113.7	0.798	0.618	3.88	0.599
WNC_1000	1170.3	1045.9	124.4	0.786	0.601	3.88	0.573

*SSA* (specific surface area), *S*_micro_ (micropore surface area), *S*_meso_ (mesopore surface area), *V*_p_ (total pore volume), *V*_micro_ (micropore volume obtained from the DFT method), *D*_meso_ (average mesopore diameter obtained from the BJH method), and *D*_micro_ (average micro diameter obtained from the DFT method) were obtained from the analysis of nitrogen sorption isotherms.

**Table 2:** Comparison of specific capacitance of the Washnut carbon electrode with other biomass-derived nanoporous carbon electrodes.

Biomass	Electrolyte	Current Density/Scan Rate	Specific Capacitance (F g^−1^)	Reference
Washnut	1 M H_2_SO_4_	1 A g^−1^	225.1	This work
Bio-decomposited product (Humic acids)	6 M KOH	0.05 A g^−1^	209	[26]
Cotton	3 M KOH	0.3 A g^−1^	221.7	[29]
Bamboo	1 M H_2_SO_4_	5 mV s^−1^	256	[31]
Corn cob	0.5 M H_2_SO_4_	0.5 A g^−1^	210	[39]
Lapsi seed	1 M H_2_SO_4_	1 A g^−1^	284	[46]
Beech (Fagus sylvatica)	1 M KOH	20 mA g^−1^	133	[56]
